# First Draft Genome of the Trypanosomatid *Herpetomonas muscarum ingenoplastis* through MinION Oxford Nanopore Technology and Illumina Sequencing

**DOI:** 10.3390/tropicalmed5010025

**Published:** 2020-02-13

**Authors:** Claudia M. d’Avila-Levy, Bertrand Bearzatto, Jérôme Ambroise, Raphaël Helaers, Anzhelika Butenko, Vyacheslav Yurchenko, Karina A. Morelli, Helena L. C. Santos, Pascal Brouillard, Philippe Grellier, Jean-Luc Gala, Miikka Vikkula

**Affiliations:** 1 Coleção de Protozoários, Laboratório de Estudos Integrados em Protozoologia, Instituto Oswaldo Cruz, Fundação Oswaldo Cruz, Rio de Janeiro 21040-360, Brazil; kmorelli@ioc.fiocruz.br (K.A.M.); helenalucias@ioc.fiocruz.br (H.L.C.S.); 2 de Duve Institute, University of Louvain, B-1200 Brussels, Belgium; raphael.helaers@uclouvain.be (R.H.); pascal.brouillard@uclouvain.be (P.B.); miikka.vikkula@uclouvain.be (M.V.); 3 Centre de Technologies Moléculaires Appliquées, Université Catholique de Louvain, B-1200 Brussels, Belgium; bertrand.bearzatto@uclouvain.be (B.B.); jerome.ambroise@uclouvain.be (J.A.); jean-luc.gala@uclouvain.be (J.-L.G.); 4 Institute of Parasitology, Biology Centre, Czech Academy of Sciences, 37005 České Budějovice (Budweis), Czech Republic; rolando24@yandex.ru; 5 Life Science Research Centre, Faculty of Science, University of Ostrava, 71000 Ostrava, Czech Republic; vyacheslav.yurchenko@osu.cz; 6 Martsinovsky Institute of Medical Parasitology, Sechenov University, 119435 Moscow, Russia; 7 Instituto de Biologia Roberto Alcântara Gomes, Departamento de Ecologia, Universidade do Estado do Rio de Janeiro, Rio de Janeiro 20550-900, Brazil; 8 Unité Molécules de Communication et Adaptation des Microorganisme (UMR 7245 CNRS MCAM), Muséum National d’Histoire Naturelle, Sorbonne Universités, 75005 Paris, France; grellier@mnhn.fr

**Keywords:** genome assembly, monoxenous trypanosomatids, insect trypanosomatids, Trypanosomatidae, whole genome

## Abstract

Here, we present first draft genome sequence of the trypanosomatid *Herpetomonas muscarum ingenoplastis*. This parasite was isolated repeatedly in the black blowfly, *Phormia regina*, and it forms a phylogenetically distinct clade in the Trypanosomatidae family.

## 1. Introduction

The family Trypanosomatidae encompasses parasites of vertebrates, invertebrates, or plants [1]. Chagas disease, leishmaniasis, and human African trypanosomiasis are human diseases caused by *Trypanosoma cruzi*, *Leishmania* spp. and *Trypanosoma brucei* sensu lato, respectively [2]. These parasites affect about 22 million people worldwide and alternate their life cycle between an insect vector and a mammalian host [3]. Therefore, the research is concentrated in these disease-inflicting parasites, however, the largest biodiversity of this family is among trypanosomatids that usually infects insects as the single host [4,5,6]. *Herpetomonas muscarum ingenoplastis* was isolated and described by Rogers and Wallace in 1971 [7]. This parasite was capable of infecting flies from nine different genera, with *Phormia* being the most prevalent genus. In artificial infections, it demonstrates high host specificity towards *Phormia regina* [7], which is a Palearctic fly found in North America and Northern Europe, also known as ”black blow fly” which plays a key role in the ecosystem via carrion decomposition and nutrient recycling [8]. 

A BLAST analysis of the single available sequence of *H. muscarum ingenoplastis* (18S rRNA gene, GenBank Acc. number KX901631) revealed that it does not cluster with any other member of the genus *Herpetomonas*. Instead, its closest phylogenetic relatives (Trypanosomatidae spp. MCC-01, MCC-02, MCC-03, GMO-05, D44-1, G42, PNG60, and MCZ-14) form a separate group on the phylogenetic tree of trypanosomatids [9,10]. Here, we sequenced the whole genome of *H. muscarum ingenoplastis* combining MinION and Illumina. 

## 2. Results and Discussion

The Illumina sequencing yielded 100,372,731 reads, out of which 89.61% presented a Phred Q score of 30 or higher, and a mean quality score of 37.55. Regarding the MinION sequencing, the starting DNA presented a good quality with a DNA Integrity Number (DIN) of 9.1. After shearing, the majority of DNA (90% of the total) was composed of fragments from 3208 bp to 46,456 bp, with an average size of 10,112 bp. Subsequently, a one-dimensional (1D) sequencing library was run for approximately 43 h in a flow cell, generating a total of 2,402,163 reads. After basecalling, 88% of the total reads passed the mean quality score threshold of 7. The ones that passed the filter had a N50 of 6514, with 2637 reads longer than 20 kb, whereas the longest read was 54.8 kb.

**Figure 1:**
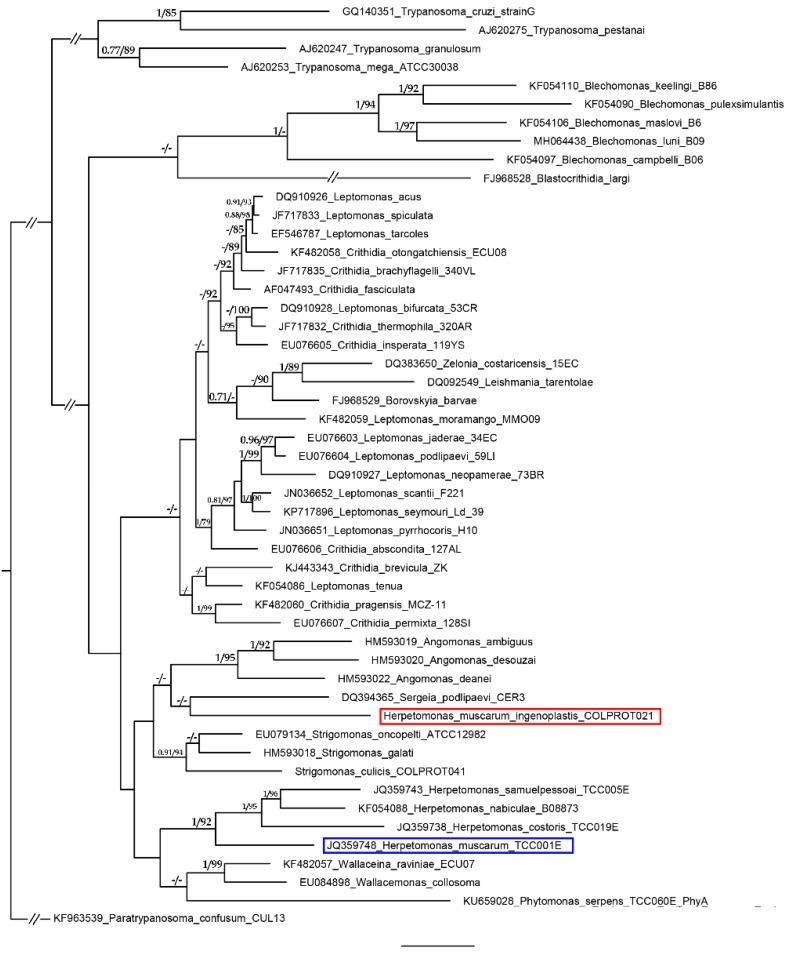
Phylogenetic analysis by ML and BI of *H. muscarum ingenoplastis*. The tree is based on the partial sequences of gGAPDH from COLPROT021 and GenBank sequences. The numbers at the top of each node denote Bayesian posterior probability and maximum likelihood bootstrap values. Dashes (-) indicate bootstrap support below 70% or different topology. The tree was rooted with the sequences from *Paratrypanosoma confusum*. Double-crossed branches are at 50% of their original lengths. The scale bar denotes the number of substitutions per site.

The assembly generated using the MinION reads in Canu consisted of 340 contigs, which were polished by the Illumina data using PILON (Appendix A). It resulted in a genome size of 35.09 Mb with an N50 of 375,483 bp, and G + C content of 53.73%. The average coverages were 428X (MinION) and 270X (Illumina). The automated annotation revealed a total of 8619 genes (Table S1 in Supplementary Materials), including putative mitochondrial proteins. The draft genome was aligned to *H. muscarum* reference genome (GCA_000482205.1) by LastZ (v. 1.04.00) revealing that only 1.5% of the latter presented an identity of 80% or higher with the draft genome. The analysis of the gGAPDH gene, widely used in barcoding and taxonomic studies [6], revealed an identity of 85% over 713 nucleotides between *H. muscarum ingenoplastis* and *H. muscarum*. The maximum likelihood (ML) and Bayesian inference (BI) phylogenetic trees reconstructed with gGAPDH were generally in agreement with the described phylogeny of the group [11] (Figure 1) and indicated that this isolate is phylogenetically distant from all described trypanosomatids, and therefore must be assigned to a new genus, as previously suggested [9,10]. 

The genomes in public database are unevenly distributed among the Trypanosomatidae family and the vast majority are concentrated in *Leishmania* and *Trypanosoma* genera (more than 50). There are five *Crithidia* spp. genomes, which are mainly used as models for biochemical, molecular, and cellular biology phenomena. There are five genomes available from representatives of the Strigomonadinae subfamily, which has attracted attention from researchers due to the possibility of deepening the understanding on endosymbiosis [12]. There are three genomes from *Phytomonas* spp. that have driven research because of the phytopathogenicity of some species [13]. Then, among the formally described genera of the family (more than 20), there are two *Leptomonas* spp. genomes, and one genome for *Paratrypanosoma*, *Endotrypanum, Blechomonas, Lotmaria,* and *Herpetomonas*. Therefore, expanding the diversity of representatives of the family with whole genome sequences would help to elucidate the phylogeny, unveil hidden biodiversity, and pinpoint specific features of the genomes and cell biology of poorly studied taxa. Particularly, *H. muscarum ingenoplastis* attracted our attention due to old reports on its exquisite cell biology, that is, the presence of double-flagellate promastigotes [7]. In the fast-changing field of long-read DNA sequencing, the Fiocruz Protist Collection decided to provide full genomic sequences of reference strains, as a strategic decision to boost science and promote Culture Collections [14].

## 3. Materials and Methods 

*H. muscarum ingenoplastis* is cryopreserved at Fiocruz Protist Culture Collection (COLPROT) (http://colprot.fiocruz.br), voucher number COLPROT-021. This specimen is also available at the American Type Culture Collection (ATCC 30259). Flagellates were grown in a biphasic medium NNN/LIT (Novy-MacNeal-Nicolle/Liver Infusion Tryptose) supplemented with 10% fetal bovine serum. The genomic DNA was extracted using PureLink Genomic DNA mini kit (Invitrogen) from cells in the late logarithmic phase of growth. DNA quality control was performed by measuring the absorbance at 260/230, concentration was determined using Qubit, and DNA integrity was analyzed by 0.8% agarose gel electrophoresis and using an Agilent 2200 Tapestation system with the Genomic DNA Screen Tape assay. Genome sequencing was performed using Illumina TruSeq DNA PCR-Free kit on Illumina HiSeq 4000 platform with 2 × 100 paired-end reads. Sequence quality metrics were assessed using FastQC (http://www.bioinformatics.babraham.ac.uk/projects/fastqc/). 

The long reads were obtained using the ONT MinION sequencer on FLO-MIN106 R9v flow cells. We prepared the library using the 1D Genomic DNA by ligation (SQK-LSK108) protocol. Briefly, high molecular weight DNA (1.3 μg) was sheared with a g-TUBE (Covaris) to an average fragment length of 8 Kb. The sheared DNA was repaired using the FFPE Repair mix (New England Biolabs), polished and an A overhang was added with NEBNext End Prep Module (New England Biolabs). Subsequently, adapters (Adapter Mix AMX1D) were ligated using the Blunt/TA Ligase Master Mix (New England Biolabs). Between each step, DNA was cleaned using Ampure XP beads (Beckman Coulter) in a 1:1 proportion. The final library was loaded on the MinION flow cell and monitored by MinKNOW software (version 1.15.1) during a 48 h sequencing time. The generated reads were basecalled, in real time, and assembled using Canu v1.4 [15]. The assembly was corrected with the Illumina data using PILON [16]. The final generated assembly was assessed by QUAST (quality assessment tool for genome assemblies) [17] in Icarus genome browser [18]. The Companion webtool (https://companion.sanger.ac.uk/) was used for gene prediction and annotation, and *Leishmania major* as a reference genome [19]. For the phylogenetic inference, gGAPDH was PCR-amplified from gDNA, sequenced, and deposited in GenBank under the accession number KX901490.1, as described elsewhere [20]. Subsequently, gGAPDH sequences were aligned using multiple sequence alignment with high accuracy and high throughput (MAFFT) online server and manually refined in BioEdit [21]. To identify the phylogenetic position of the isolate, phylogenetic trees were created using *Paratrypanosoma confusum*, as the outgroup [11]. Phylogenetic trees were constructed using two probabilistic methods, ML and BI, which were based on GTR + G substitution model, according to the Akaike Information Criterion and Bayesian information criterion (BIC) using Jmodeltest [22]. The ML tree was created in METAPIGA v2.0 [23] and BI in MrBayes v3.2 [24]. By the analysis of 1000 replicates and the MCMC algorithm, with four chains, the bootstrap values were determined for the ML and BI, respectively. For each 1000 generations, chains were sampled out of a total of 10^7^ generations. Convergence was evaluated by the mean standard deviation of split frequencies that were lower than the recommended values (<0.01). For each dataset, the first quarter of the selected trees was excluded as burn-in, and the nodal support and consensus tree topology were assessed from the remaining samples as posterior probability values.

## Supplementary Materials

The following are available online at https://www.mdpi.com/2414-6366/5/1/25/s1, Table S1: Putative protein list based on the automated annotation by Companion, using Leishmania major as reference.

## Appendix A

Data access

This Whole Genome Shotgun project has been deposited at DDBJ/ENA/GenBank under the accession VFSE00000000. The version described in this paper is version VFSE00000000.1.
